# Pine wilt disease detection algorithm based on improved YOLOv5

**DOI:** 10.3389/fpls.2024.1302361

**Published:** 2024-04-18

**Authors:** Zengjie Du, Sifei Wu, Qingqing Wen, Xinyu Zheng, Shangqin Lin, Dasheng Wu

**Affiliations:** ^1^ College of Mathematics and Computer Science, Zhejiang A&F University, Hangzhou, China; ^2^ Key Laboratory of State Forestry and Grassland Administration on Forestry Sensing Technology and Intelligent Equipment, Hangzhou, China; ^3^ Key Laboratory of Forestry Intelligent Monitoring and Information Technology of Zhejiang Province, Hangzhou, China; ^4^ Wucheng Nanshan Provincial Nature Reserve Management Center of Zhejiang Province, Jinhua, China

**Keywords:** pine wilt disease, unmanned aerial vehicle, deep learning, YOLOv5, SimAM-ASFF

## Abstract

Pine wilt disease (PWD) poses a significant threat to forests due to its high infectivity and lethality. The absence of an effective treatment underscores the importance of timely detection and isolation of infected trees for effective prevention and control. While deep learning techniques combined unmanned aerial vehicle (UAV) remote sensing images offer promise for accurate identification of diseased pine trees in their natural environments, they often demand extensive prior professional knowledge and struggle with efficiency. This paper proposes a detection model YOLOv5L-s-SimAM-ASFF, which achieves remarkable precision, maintains a lightweight structure, and facilitates real-time detection of diseased pine trees in UAV RGB images under natural conditions. This is achieved through the integration of the ShuffleNetV2 network, a simple parameter-free attention module known as SimAM, and adaptively spatial feature fusion (ASFF). The model boasts a mean average precision (mAP) of 95.64% and a recall rate of 91.28% in detecting pine wilt diseased trees, while operating at an impressive 95.70 frames per second (FPS). Furthermore, it significantly reduces model size and parameter count compared to the original YOLOv5-Lite. These findings indicate that the proposed model YOLOv5L-s-SimAM-ASFF is most suitable for real-time, high-accuracy, and lightweight detection of PWD-infected trees. This capability is crucial for precise localization and quantification of infected trees, thereby providing valuable guidance for effective management and eradication efforts.

## 1 Introduction

Pine wilt disease (PWD) is a disease caused by wood pathogens carried by the pinewood nematodes. It is characterized by a brief incubation period, strong infectivity, and the ability to kill pine trees within just 40 days after infection. The entire pine forests can be completely destroyed within 3-5 years from the initial outbreak of a single pine tree. PWD stands out as one of the most dangerous and destructive diseases affecting forest ecosystems in China and even the world (Li et al., 2023). Pinewood nematode is native to North America and is less harmful to native pine plants. However, after invading Asia and Europe (Kim et al., 2018, 2020; Lim et al., 2022), the PWD has had disastrous effects on pine trees in countries such as Japan, Korea, China, Portugal, and Spain (Ohsawa and Akiba, 2014; Wang and Zhang, 2015; Wang et al., 2022). Since 1982, when PWD was first discovered in China, the disease has caused the death of hundreds of millions of pine trees, with an annual average of about 27 million dead trees, making it the biggest killer of China’s pinewoods (Yang et al., 2014).

However, there is no effective eradication means for PWD, so timely detection and isolation of diseased pine trees have become crucial for controlling its spread. Pine trees infected with PWD dehydrate and eventually die due to leaf stem blockage, causing their green pine needle-like leaves to discolor (turning yellow, yellow-brown, or red) and appear lifeless. The above characteristics provide important auxiliary information for the rapid identification of pine wilt diseased trees. Currently, the detection of PWD primarily relies on field investigation by forest protection personnel to collect data on the specific coordinates and locations of individual diseased pine trees. This approach is not only inefficient but also hindered by terrain and landscape challenges, making it difficult to obtain real-time information of pine wilt diseased trees. Fortunately, with the development of unmanned aerial vehicle (UAV) technology, high-definition cameras mounted on UAVs are now capable of capturing images with centimeter-level resolution. Additionally, when UAVs capture images of wild forests, human operators have the flexibility to select sunny weather conditions to photograph the study area, effectively reducing the mitigating interference from clouds, rain, and snow. It can quickly collect images of areas that cannot be reached by humans on a large scale (Blackman and Yuan, 2020). UAVs have been increasingly used for monitoring PWD (Yu et al., 2021; Sun et al., 2022). Kim and Deng et al. (Kim et al., 2017; Deng et al., 2020) used UAV remote sensing technology to identify and locate pine wilt diseased tree and found that the disease is not related to the species of pine trees. Liu Xialing et al. (Liu et al., 2018). utilized a multi-template recognition method to identify infected pine trees based on drone images at different stages of PWD. Lee and Zhang et al. (Lee et al., 2019; Zhang et al., 2022b) combined UAV remote sensing technology with artificial neural networks (ANN) and support vector machines (SVM) to identify pine wilt diseased trees in complex terrain. Tao Huan et al. (Tao et al., 2019). acquired RGB images by UAV and used the HSV threshold method to identify discolored pine wilt diseased trees. Qin et al. (Qin et al., 2021). proposed a method to identify PWD based on UAV multispectral remote sensing images and a new network called SCANet, which retains spatial and contextual information and reduces the occurrences of false detections and missed detections. Xu Xinluo et al. (Xu et al., 2020). utilized convolutional neural networks (CNN) to monitor the research area of PWD based on UAV hyperspectral data, verifying the high accuracy of target detection in the UAV images of PWD.

The aforementioned studies extensively utilized UAVs to acquire multispectral, hyperspectral, RGB images, and other pertinent data from the designated study areas. These studies employed diverse machine learning algorithms to precisely detect PWDs. The efficient coverage of vast areas by UAVs, coupled with the application of sophisticated algorithms and models, significantly enhanced the accuracy and reliability of disease identification.

In recent years, deep learning (DL) algorithms have been widely applied in pattern recognition and have achieved great successes (Jermsittiparsert et al., 2020). Compared with traditional machine learning methods, DLs exhibit remarkable superiority in feature extraction. They can automatically learn and extract high-level, abstract features from raw data without requiring extensive manual feature engineering. This automated feature extraction capability significantly simplifies the data preprocessing workflow and reduces the reliance on expert feature design. Additionally, deep learning models, through complex combinations and transformations of multilayer neural networks, can capture deeper relationships and patterns within the data. This allows deep learning algorithms to excel in complex tasks such as image recognition, speech recognition, and natural language processing, achieving numerous breakthrough results. Therefore, DL networks, including YOLO (You Only Look Once), Faster R-CNN (Region-based Convolutional Neural Networks), and SSD (Single Shot Multibox Detector), have increasingly found their applications in disease recognition and target classification (Wang et al., 2021; Zhang et al., 2021; Xie and Hu, 2022; Xu et al., 2023). The combination of UAV (Unmanned Aerial Vehicle) remote sensing technology with these DL networks has emerged as a prominent trend in monitoring PWD (Pine Wilt Disease), yielding significant progress and achievements. For instance, Hu et al. (Hu et al., 2022). effectively integrated DDYOLOv5 with the ResNet50 network, introducing efficient channel attention and hybrid dilated convolution modules. This approach achieved remarkable results in detecting and classifying PWD in UAV remote sensing images. Similarly, Zhou et al. (Zhou et al., 2022). proposed a Multi-band Image Fusion Infection Pine Detection (MFTD) detector, which accurately pinpointed PWD using a combination of UAV visible light and multispectral images, particularly in its early stages. Specifically, the average precision values (AP@50) were 87.2%, 93.5%, and 84.8% for early, middle, and late stages on the KP dataset and 81.2%, 92.9%, and 86.2% on the CP dataset. Although MFTD achieved a high average precision in detecting PWD, the model’s parameter count was 413.4MB with an FPS of 35.6. However, despite these advancements, practical applications still face several challenges. One such challenge is the relatively large size of these models, which can hinder their deployment in resource-constrained environments. Additionally, as the accuracy of these networks improves, there is often a trade-off with real-time capabilities, as model complexity increases. This highlights the need for further research in developing more efficient and lightweight models that can maintain high accuracy while preserving real-time performance.

To enhance the utility of DL networks and UAV remote sensing technology in monitoring and managing PWD and similar diseases, it is crucial to address the aforementioned challenges.

When utilizing UAVs to capture images for the identification of trees infected with PWD, the preferred approach is to execute the model directly on a mobile terminal device, such as a tablet computer. This facilitates prompt navigation to suspected infected trees for on-site verification and remedial action. However, compared to PCs and servers, mobile devices typically possess limited computing and storage capabilities. Among the remote sensing devices mounted on UAVs, optical RGB cameras offer a cost-effective solution for data acquisition while facilitating easier processing and analysis on mobile devices when compared to multispectral cameras or radar sensors. Simultaneously, we need to better balance performance between accuracy, efficiency, and real-time capabilities when developing algorithms for predicting PWD.

This study aims to find a lightweight and high-precision DL algorithm and attempts to improve it to be more suitable for use on RGB images captured by UAVs to identify PWD-infected trees. Specifically, leveraging RGB images of forests obtained via UAVs, we conducted a comparative analysis of Faster R-CNN, SSD, and YOLO algorithms, ultimately selecting a lightweight and efficient algorithm as our base model. Subsequently, we refined its backbone network, augmented target feature representation through the introduction of a spatial attention mechanism, and enhanced both the recognition accuracy of PWD-infected trees and the model’s robustness against various disturbances by incorporating multi-scale feature fusion and spatial context enhancement through adaptively spatial feature fusion.

## 2 Materials and methods

### 2.1 Study area

**Figure 1:**
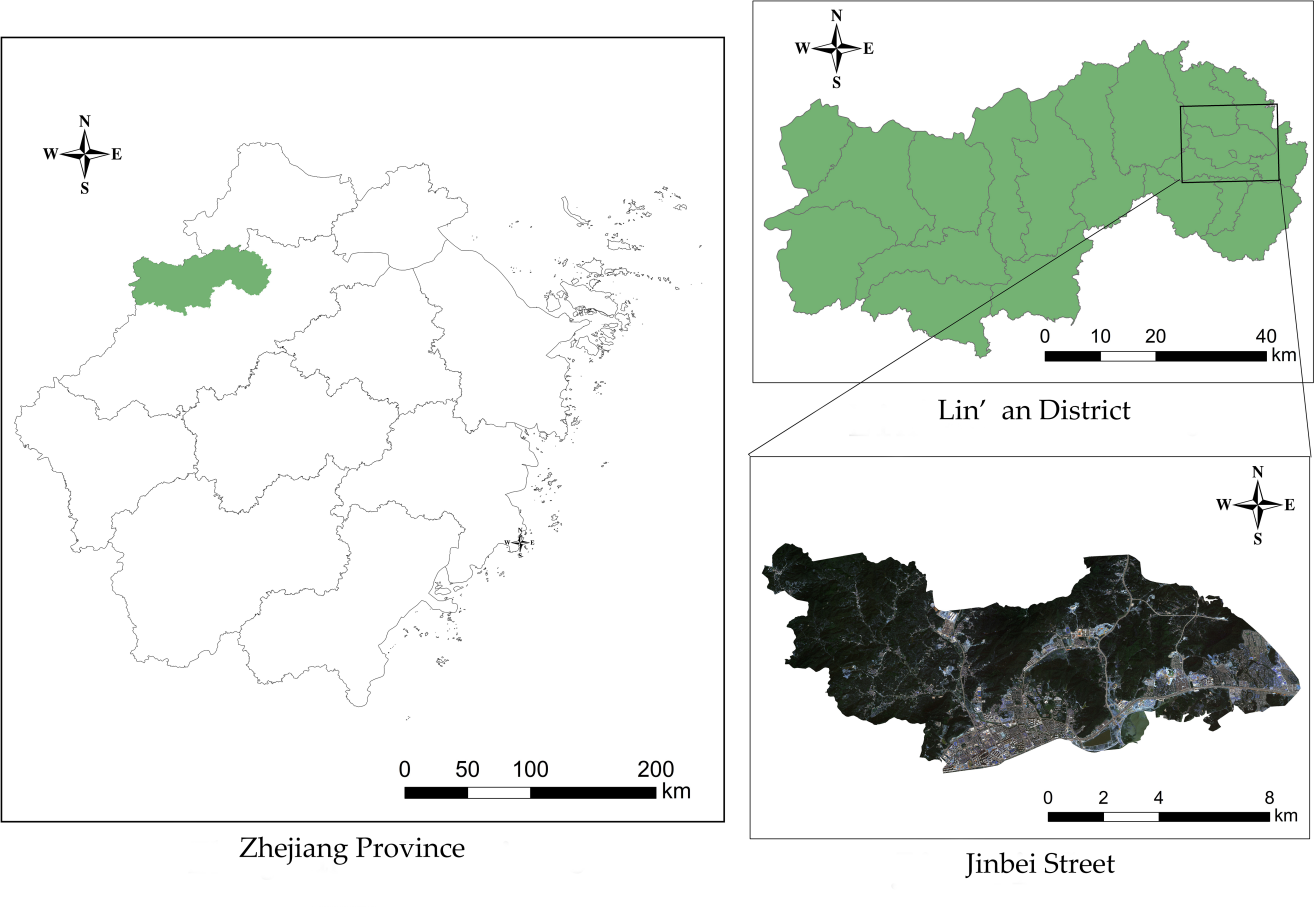
Location of the study area (Jinbei Street).

The study area (**Figure 1**), Jinbei Street, is in Lin’an District, Hangzhou City, Zhejiang Province, China (118°51’~119°52’E, 29°56’~30°23’N). Lin’an was designated as a PWD epidemic area by the former State Forestry Administration in 2008. Admittedly, through more than ten years of prevention and control of PWD, Lin’an District has preserved the important landscapes of pine forests. However, there are still some problems such as insufficient attention and low accuracy of the census in some towns and streets, resulting in the serious PWD in some areas. Jinbei Street is located in the eastern of Lin’an District, with an administrative area of 81.54 square kilometers (122,310 mu) and with Pinus massoniana as the main tree species of Pine. There are 689 sub-compartments of pine, with an area of 2,343.3 hectares proportioning 28.28% of the administrative area. Pinewoods resources play a very important role in the construction of ecological environment as well as economic development in this area. Due to the rapid spread of PWDs, Jinbei Street has become one of the serious epidemic areas in Lin’an District.

### 2.2 Data collection

The UAV remote sensing data was captured by the DJI Genie 4 PRO RTK multi-rotor UAV (Da Jiang Innovations, Inc., Shenzhen, Guangdong, China) equipped with an FC6310R aerial camera. The technical parameters of the equipment are shown in **Table 1**.

**Table 1:** DJI Genie 4 PRO RTK multi-rotor UAV and technical parameters of aerial camera.

Parameter Name	Parameter Value
Name of UAV	DJI Genie 4 PRO RTK
Aerial Camera	FC6310R
Sensor Size	13.2mm×8.8mm
Lens focal length	8.8mm

**Figure 2:**
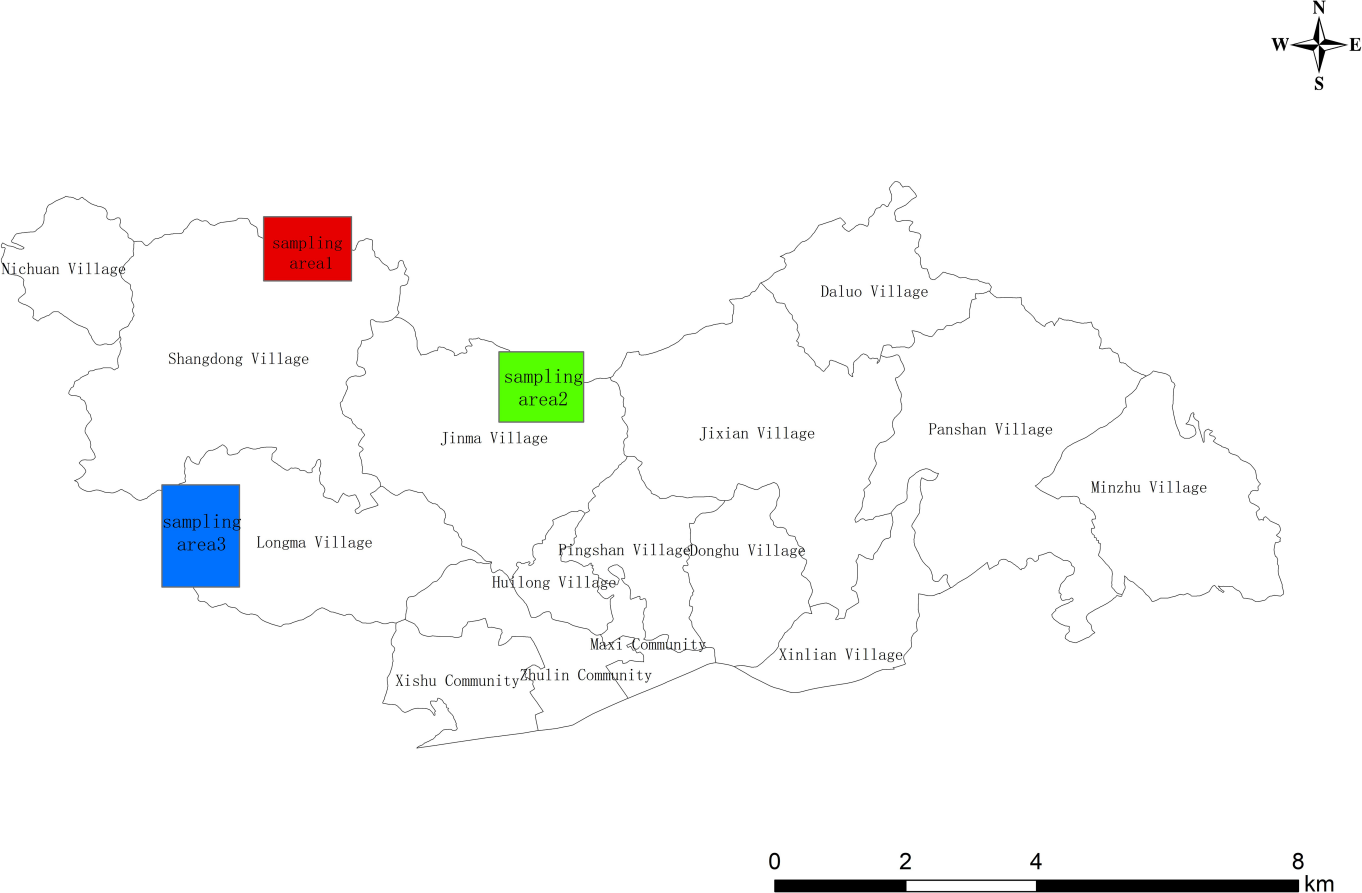
Accuracy verification sampling area.

The PWD occurs annually from May to October. The nematodes enter the pine tree through wounds and reproduce in large numbers. The symptoms are evident in August and September and ultimately cause a severe infection of the tree, which wilts and dies around October. In order to effectively verify the remote sensing image identification results, the distribution of pine wilt diseased trees in the study area was roughly identified through the analysis of Pléiades satellite images in October. Furthermore, verification sampling areas were set up in Shangdong village, Jinma village, and Longma village respectively (as shown in **Figure 2**). Subsequently, within one week after acquiring satellite images, UAV images of the affected area were captured, providing crucial baseline data for verifying the accurate identification of pine wilt diseased trees based on the DL algorithm.

To better adapt to the terrain and vegetation characteristics of the target area, the parallax overlap being about 70%, the heading overlap being 80% and a fixed flying altitude of 300 meters were presented by the Pix4D Mapper software (Pix4D Company, Switzerland). The images were captured between 12:00 and 14:00 when sunlight is sufficient and it can effectively avoid interference by oblique sunlight. Eventually, the images were geometrically corrected and stitched together by DJI Terra software (Da Jiang Innovations, Inc., Shenzhen, Guangdong, China) to produce an orthophoto map of the area.

### 2.3 Dataset production

The orthophoto map was further segmented into 999 RGB images with a size of 5472*3648 pixels. However, to facilitate processing on resource-constrained devices like mobile terminal devices, these images were further resized to 1824x1824 pixels using the batch image cropping tool IrfanView (available at https://www.irfanview.net, version 4.62). Additionally, images lacking PWD-infected pine trees or those of poor quality (blurred images where even experienced professionals struggle to visually identify PWD infection) were discarded, resulting in a refined dataset of 1041 images.

Subsequently, guided by the experts of forest disease, our study made use of LabelImg, a python-based annotation tool retrieved from https://github.com/heartexlabs/labelImg on April 16, 2023, to systematically annotate the image datasets in compliance with the Pascal VOC data label storage format. The dataset was then divided at random into a training set and a testing set, following a 9:1 ratio.

**Figure 3:**
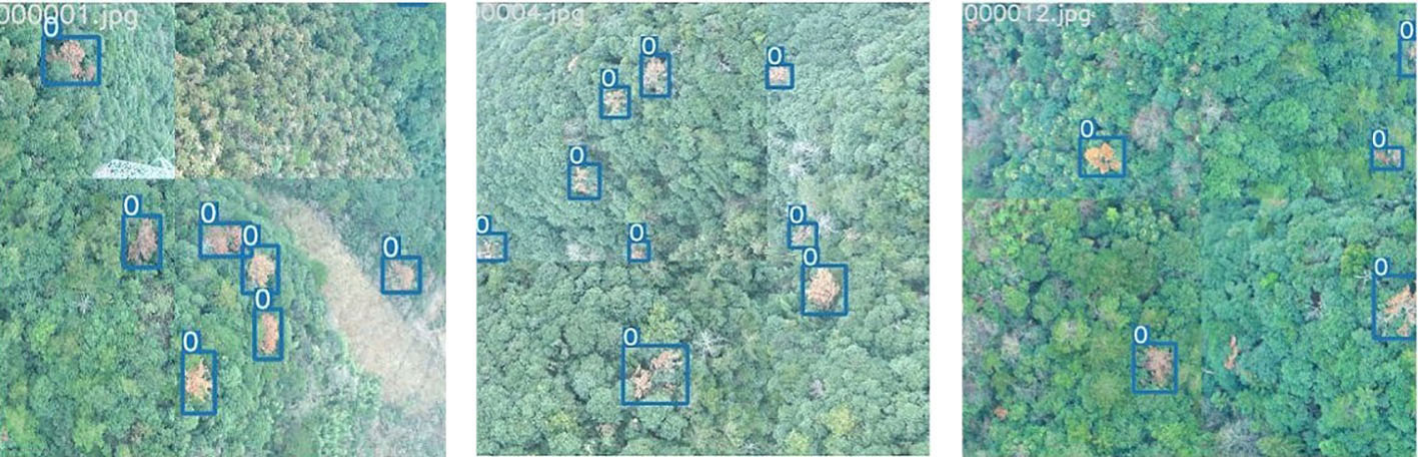
Training samples after data augmentation.

After that, we employed Mosaic Data Augmentation from YOLOv5 to enhance the training set data. This technique merges four distinct images into one composite image, introducing randomness in scaling, cropping, and image arrangement. This approach significantly enhances the diversity of object sizes, backgrounds, and perspectives within the dataset. By leveraging Mosaic Data Augmentation, YOLOv5 is able to effectively expand its training dataset, ultimately leading to improved model generalization and overall performance. **Figure 3** visually illustrates training samples after data augmentation, where “0” denotes instances of “PWD-infected tree”.

Furthermore, to achieve a more comprehensive evaluation of the generalization ability, this study employed a 10-fold cross-validation method to evaluate the accuracy of the model.

### 2.4 Experimental environment and parameters

The experimental environment was constructed on the AutoDL cloud server, leveraging an Intel Xeon processor (Skylake, IBRS) with a 10-core CPU and 56 GB RAM. Simultaneously. The software configuration comprised of Ubuntu 20.04 as the operating system, Python 3.8 as the development language, and PyTorch 1.10.0 as the development framework. During the training, the input image size was set to 640×640 pixels, with a freeze training strategy. Initially, the backbone network parameters were frozen for 100 epochs, with a batch size of 8 (simultaneous training of 8 images per batch) and a learning rate of 0.001. Subsequently, the unfreezing of network parameters were set as: epoch is 200, batch size is 4, and the learning rate is 0.0001. This freeze-unfreeze approach was expected to accelerate network training and potentially improve the generalization ability of the model.

### 2.5 Improved YOLOv5Lite-based detection method

**Figure 4:**
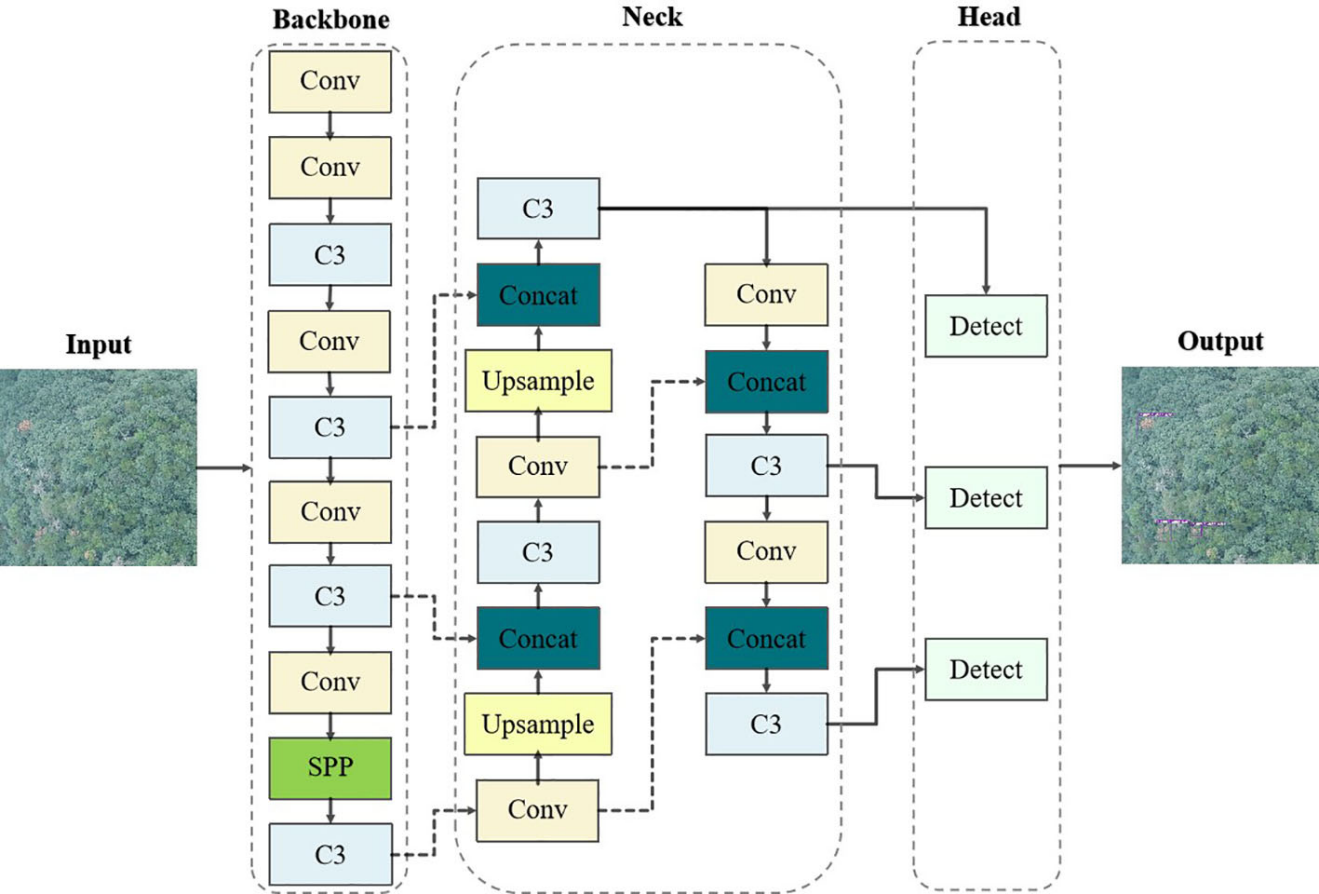
YOLOv5-Lite network structure.

In this paper, YOLOv5-Lite (as shown in **Figure 4**) was utilized as the fundamental algorithm for detecting PWD-infected trees. Compared with YOLOv5, the YOLOv5-Lite introduces innovative techniques to enhance the detection speed and reduce model parameters count, thus fulfilling the dual objectives of higher detection accuracy and stronger real-time performance.

**Figure 5:**
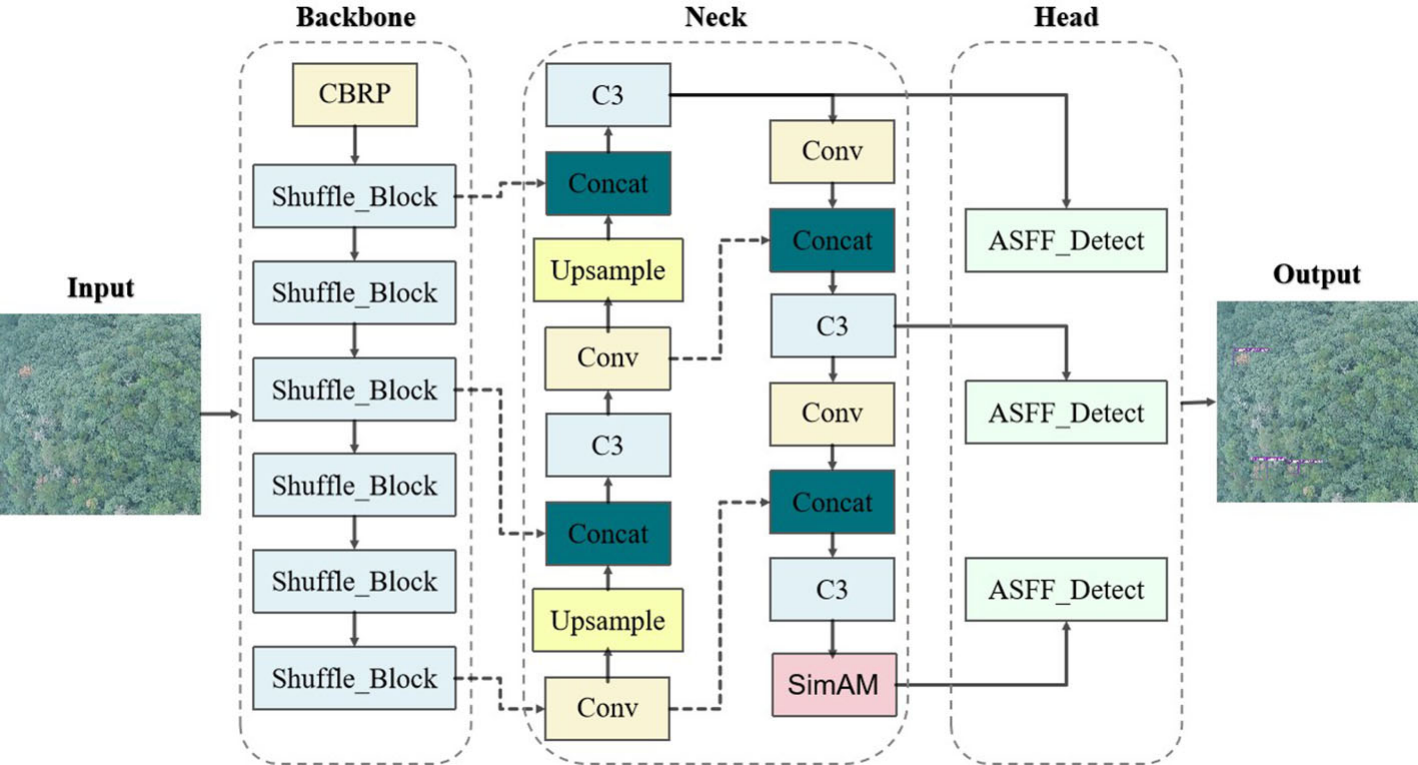
Improved YOLOv5-Lite network structure.

PWD-infected pine trees are often obscured in various complex backgrounds, covering by other trees or only appearing subtle targets. In standard YOLOv5-Lite implementations, small target features tend to diminish as the network depth increases during detection. Consequently, the detailed features of these small targets gradually disappearing in the subsequent network propagation, causing missed or false detection. Moreover, this study aims to apply the detection model to mobile devices for rapid, real-time identification. To address these challenges, we enhanced the YOLOv5-Lite by refining its model size and parameter number for increased lightness. In addition, we improved the feature extraction and fusion capability of the neck network to increase accurate. Specifically, our proposed algorithm (as shown in **Figure 5**) replaced the original CSPLocknet53 backbone with the light-weight ShuffleNetV2. It also integrated the SimAM attention mechanism into the neck network path aggregation network (PANet), and appended an adaptively spatial feature fusion (ASFF) module after PANet for improving performance.

#### 2.5.1 Lighten backbone network

Due to the computational complexity and extensive parameters inherent in the CSPDarknet53 backbone of traditional YOLOv5-Lite, it is not suitable for scenarios in resource-constrained environments such as mobile devices.

**Figure 6:**
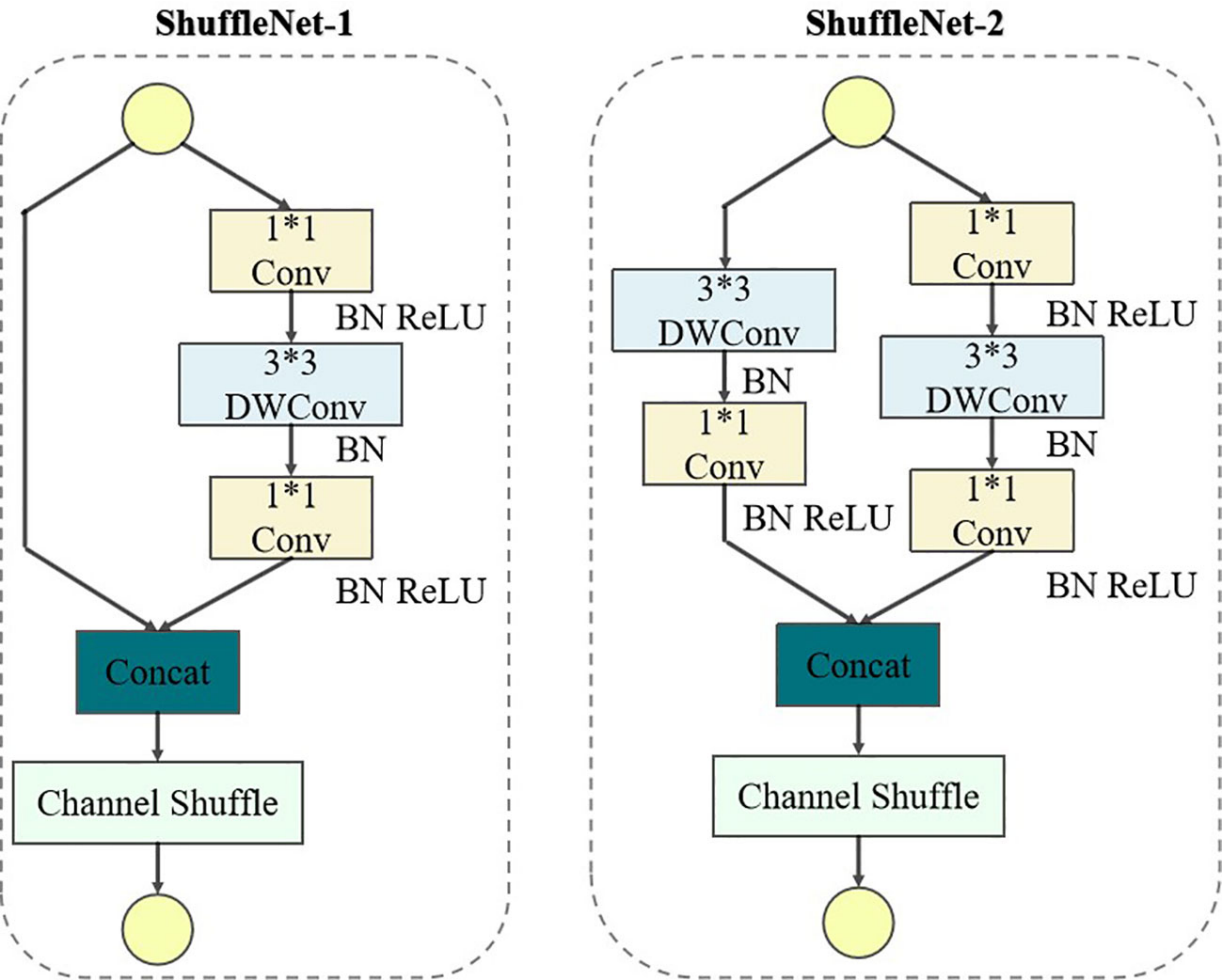
Network structure of ShufflenetV2.

ShuffleNetV2 (as shown in **Figure 6**) is a lightweight neural network that mainly uses Channel Shuffle and Pointwise Group Convolution technology to greatly reduce the computation and parameter amount of the network. By integrating ShuffleNetV2 into YOLOv5-Lite, it can significantly lighten the model, thereby accelerating its training without significantly decreasing the model performance.

In the ShuffleNet-1 unit of the ShuffleNetV2 (as shown in **Figure 6**), the input feature map is first divided into two branches, each with half the channels. The left branch preserves the feature map intact through identity mapping, while the right branch goes through three convolutions including two 1×1 ordinary convolutions and a 3×3 depthwise convolution (DWConv), both using the same input and output channel numbers. Following the convolutions, the branches are reunited through concatenation (Concat), and Channel Shuffle is employed to facilitate cross-group information exchange, ensuring thorough channel integration. Contrastingly, in the ShuffleNet-2 unit, the feature map is directly distributed across two branches, both utilizing 3×3 DWConv with a step of 2. This configuration effectively reduce the height (H) and width (W) of the feature map, thereby reducing the amount of computational overhead. The outputs from branches are then concatenated by the Concat operation, doubling the channel count while without changing the network parameter amount significantly. Finally, channel shuffling is utilized to exchange information across channels.

In the traditional YOLOv5-Lite backbone network, direct point-to-point convolution between the image and the filter results in a high computational complexity. However, The DWConv introduce multiple convolution kernels for multiple input channels to reduce the number of the parameters which is calculated by (Equation 1).


(1) $$C=D_{K}\times M\times N\times D_{F}$$

(2) $$C^{\prime }=D_{K}\times M\times D_{F}+M\times N\times D_{F}$$

(3) $$\frac{C^{\prime }}{C}=\frac{1}{N}+\frac{1}{D_{K}}<1$$

Where, ${\text{D}}_{\text{K}}$ denotes the size of the convolution kernel, M is the number of input channels, N is the number of convolution kernels, and D_F_ is the size of the input image. The DWConv decomposes a complete convolution block into two convolution blocks by first using channel-by-channel convolution on M input channels, and then adjusting the number of output channels by pointwise convolution based on N 1×1 convolution kernels. The resulting parameter count (C′) is determined by (Equation 2). By comparing the relationship between C and C′ [as shown in (Equation 3)], it was found that the improved network can significantly reduce the number of parameters, boosting processing efficiency.

However, excessive depth in the network introduced by depth-separable convolution can lead to gradient dispersion, impeding model convergence. Thereby, it is necessary to introduce the residual structures by directly backpropagation of errors from the output layer to the input layer to avoid gradient disappearance.

#### 2.5.2 Enhanced feature extraction

**Figure 7:**
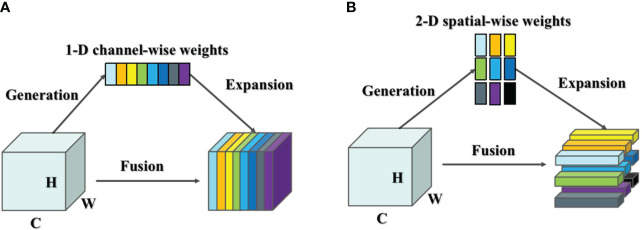
**(A)** Channel Attention mechanism and **(B)** Spatial Attention mechanism.

YOLOv5-Lite treats the target detection process as a regression task. However, it does not distinguish well between the foreground and background regions in the input image, potentially leading to missed or incorrect detections. This challenge arises due to the limited number of occupied pixels by the foreground target and the presence of a complex background, which can obscure crucial information about the target. To address this issue, this study enhances the feature representation of the target by introducing an attention mechanism. The attention mechanism can be broadly categorized into two types: spatial attention mechanism and channel attention mechanism (as shown in **Figure 7**) (Mo et al., 2023).

The spatial attention mechanism focuses on different spatial locations in the feature map. To detect target, the feature map can be viewed as a matrix consisting of a series of feature vectors extracted from the input image. The spatial attention mechanism adaptively adjusts the weights of the features at different locations by calculating the importance of each spatial location to enhance the attention to the critical regions of the target object. On the other hand, the channel attention mechanism considers different channels in the feature map, representing various feature dimensions. It adaptively weights these channels based on their importance, thereby enhancing attention to significant features of the target object. These two attention mechanisms correspond exactly to feature-based attention and spatial-based attention in the human brain, offering a biologically inspired approach to improving target detection accuracy.

Most attention mechanism modules inherit the principle of action into each block to improve the output from previous layers. This process typically generates one- or two-dimensional weights, either along the channel dimension or spatial dimension, treating neurons uniformly in each channel or spatial location. The channel attention mechanism is a 1-D attention mechanism that distinguishes between channels while treating all locations equally, thereby enhancing the feature representation of the occluded target. Conversely, the spatial attention mechanism is a 2-D method that emphasizes different locations of the feature map that are relevant to the current task, while considering all channels equally.

In human brain, spatial attention and channel attention always coexist to jointly facilitate information selection during visual processing. The existing attention mechanisms, such as CBAM and SimAM, combine spatial attention mechanisms and channel attention mechanisms in parallel or serial fashion.

In neuroscience, neurons carrying rich information often exhibit firing patterns that are distinct from neighboring neurons. To differentiate the importance of these neurons and achieve effective attention, an energy function is employed. This function determines the linear separability between the target neuron and all other neurons in the same channel (Lei et al., 2023). The final energy function is presented in (Equation 4).


(4) $$e_{t}\left(w_{t},b_{t},y,x_{i}\right)=\frac{1}{M-1}\sum_{i=1}^{M-1}{\left(-1-\left(w_{t}x_{i}+b_{t}\right)\right)}^{2}+{\left(1-\left(w_{t}t+b_{t}\right)\right)}^{2}+\gamma w_{t}^{2}$$

where t denotes calibrated neuron. The weight and bias at neuron transformation in (Equation 4) are shown in (Equation 5) and (Equation 6).


(5) $$w_{t}=\;-\frac{2\left(t-\mu_{t}\right)}{{\left(t-\mu_{t}\right)}^{2}+2\delta_{t}^{2}+2\gamma }$$

(6) $$b_{t}=\;-\frac{1}{2}\left(t+\mu_{t}\right)w_{t}$$

where μ and $\delta_{t}^{2}$ are the mean and variance of all neurons except t. μ is shown in (Equation 7) and $\delta_{t}^{2}$ is shown in (Equation 8).


(7) $$\mu_{t}=\frac{1}{M-1}\sum_{i=1}^{M-1}x_{i}$$

(8) $$\delta_{t}^{2}=\frac{1}{M-1}\sum_{i=1}^{M-1}{\left(x_{i}-u_{i}\right)}^{2}$$

Given the assumption that all pixels in a single channel follow the same distribution, we can calculate the mean and variance once and reuse them for all neurons on that channel. This approach significantly reduces computation costs by avoiding the need to iteratively calculate μ and σ for each position. Therefore, the minimum energy can be obtained by (Equation 9):


(9) $$e_{t}^{*}=\frac{4\left(\delta^{2}+\gamma \right)}{{\left(t-\varepsilon \right)}^{2}+2\delta^{2}+2\gamma }$$

The (Equation 9) indicates that a lower the energy value corresponds to a greater difference between neuron t and its neighboring neurons, signifying higher the importance. Moreover, the input features are further enhanced by (Equation 10):


(10) $$\tilde{X}=sigmoid\left(\frac{1}{E}\right)⊙X$$

where X denotes the input feature vector, E represents the energy value of each neuron. The enhanced feature vector is obtained by applying the Sigmoid function to activate E of each neuron.

The attention mechanism is a plug-and-play module that can theoretically be integrated after any feature layer, such as the backbone network or an enhanced feature extraction network. However, in the context of transfer learning, if the attention mechanism module is incorporated into the backbone network, the pre-training weights of the network may become unavailable. Therefore, in our proposed algorithm, the attention mechanism is applied specifically to the enhanced feature extraction network.

#### 2.5.3 Enhanced feature fusion

**Figure 8:**
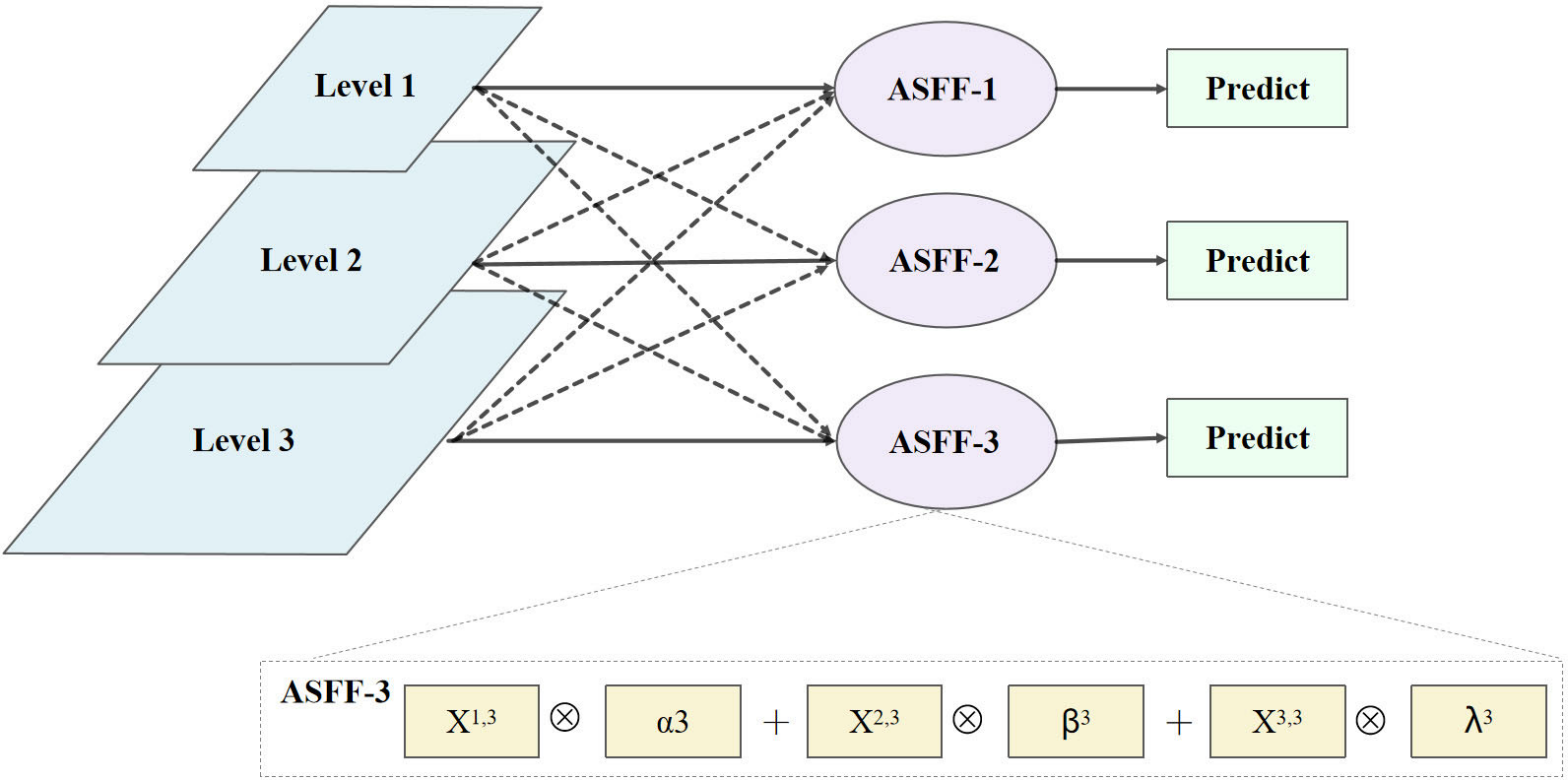
Network structure of ASFF.

Since PANet in YOLOv5-Lite doesn’t fuse multi-scale features, it struggles to fully leverage both low-level details and high-level semantic for target identification. For diseased pine tree targets with complex backgrounds and even serious occlusion, some low-level information tends to weaken the information representation capability of the upper-level features in the bottom-up transmission process. To solve this problem, ASFF (as shown in **Figure 8**) structure is introduced after PANet. Therefore, the three feature outputs of PANet are fused by adaptive learning to make full use of low-level detail information and high-level semantic information.

ASFF is a network based on adaptive spatial feature fusion. It takes the Level1, Level2, and Level3 layer feature maps output from the PANet network as input. After the feature fusion operations of ASFF-1, ASFF-2, and ASFF-3 are performed respectively, the fused feature maps of each layer are finally predicted as output. During fusion, all layers are adjusted to have the same channel size and number, and corresponding weight coefficients are calculated. Finally, the feature map is multiplied by the weight coefficient of each layer and the sum of the multiplication results is calculated to achieve feature fusion.

Taking ASFF-3 as an example, the feature fusion process is expressed as (Equation 11).


(11) $$y_{3}=\alpha_{3}\cdot X_{1\to 3}+\beta_{3}\cdot X_{2\to 3}+\gamma_{3}\cdot X_{3\to 3}$$

where ${\text{y}}_{3}$ is level-3 of the feature map; X_1→3,_ X_2→3_, and X_3→3_ denote the feature maps output when each layer feature map is adjusted to the same size and number of channels as the level-3 feature map, respectively; ${\text{α}}_{3}$ , ${\text{β}}_{3}$ , and γ_3_ are the weight coefficients learned by the three feature maps of X_1→3,_ X_2→3_, and X_3→3_, when feature fusion is performed at the level-3. The specific procedure is as follows: Firstly, perform a 1×1 convolutional operation to the three feature maps X_1→3,_ X_2→3_, and X_3→3_; Secondly, perform channel cascading operations; Thirdly, use SoftMax to calculate the weight values which are within the range of [0,1] and satisfying the constraint of ${\text{α}}_{3}+{\text{β}}_{3}+{\text{γ}}_{3}=1$ . Finally, the three feature maps are multiplied by the weight coefficients for each layer and the sum of the multiplication results is calculated to achieve feature fusion.

When adjusting the size and number of channels for each layer, the adjustment methods are different for feature fusion operations of ASFF-1, ASFF-2, and ASFF-3. As shown in **Figure 8**, the adjustment methods are down-sampling operation for ASFF-1, while up-sampling operation for ASFF-3.

Due to the different sizes of the target images in the dataset in this study, they should be resized to a uniform size in the input to the model. However, the resizing may lead to the targets smaller, which is more difficult to identify the foreground target from the complex background. Fortunately, ASFF enhances the feature information of smaller targets, thereby improving detection accuracy.

### 2.6 Evaluation index

We chose a 10-fold cross-validation method to evaluate the accuracy of the model. There are six evaluation indexes, i.e., three accuracy evaluation indexes of mean Average Precision (mAP), Recall, and F1, and three indexes to evaluate the lightweighting effect, that is, Frames Per Second (FPS), model size, and number of model parameters. mAP is one of the most important metrics in target detection to evaluate the detection accuracy performance of the model, its value is usually calculated by an Intersection over Union (IoU) with a threshold of 0.5. Recall represents the proportion of positive samples correctly predicted to all true positive samples, and F1 is the total average of model accuracy and recall, which is used to evaluate the effectiveness of the model. In the three indexes to evaluate the lightweighting effect, the larger the frames per second (FPS) transmission, the more images can be detected per second and the smoother the display. The smaller the model size, the better the lightweighting of the model. Similarly, the smaller the number of parameters, the better the lightweighting of the model. Specifically, the indicators are determined by Equations 12–16.


(12) $$IOU=\frac{A\cap^{\;}B}{A\cup^{\;}B}$$

(13) $$precision=\frac{TP}{TP+FP}$$

(14) $$Recall=\frac{TP}{TP+FN}$$

(15) $$F1=\;2\;\times \;\frac{precision\;\times \;Recall}{precision\;+\;Recall}$$

(16) $$mAP\;=\;AP\;=\;\int_{0}^{1}P(R)dR$$

Where, A and B are the measured and predicted values, respectively. “Positive” denotes a prediction that the pine trees are infected with PWD, while “negative” denotes a prediction that the pine trees are not infected with PWD. A true positive (TP) means the pine tree is actually infected with PWD and the prediction is also positive. Conversely, a false positive (FP) means the tree is not infected with PWD, but the prediction is positive. False negative (FN) is defined similarly for the case where the prediction is negative and the tree has not infected with PWD in fact.

## 3 Results

The performance metrics, including mAP, Recall, F1, FPS, Model Size, and Number of model parameters, were generated by models: Faster R-CNN, SSD, YOLOv5Lite, and YOLOv7, as well as the improved networks based on YOLOv5Lite. These results are presented in **Table 2**. In this study, YOLOv5L represented the original YOLOv5-Lite algorithm, YOLOv5L-s represented an enhanced version where the backbone network, CSPDarknet53, was substituted with the lightweight network ShuffleNetV2. YOLOv5L-s-SimAM further improved upon YOLOv5L-s by introducing the SimAM module to the last convolutional layer of the feature extraction network. Similarly, YOLOv5L-s-CBAM incorporated CBAM instead of SimAM. Finally, YOLOv5L-s-SimAM-ASFF augmented YOLOv5L-s-SimAM by adding the ASFF module to the end of PANet. Due to its optimal performance indicators, the YOLOv5L-s-SimAM-ASFF was identified as the ultimate improved algorithm proposed in this study.

**Table 2:** The performance metrics of different models.

Models	mAP(%)	Recall(%)	F1(%)	FPS	Size(MB)	Parameters	p-value
Faster R-CNN	87.45	79.96	83.49	10.56	108	137098724	**1.31e-51**
SSD	90.76	83.31	86.93	24.11	90.6	26285486	2.82e-24
YOLOv7	94.16	91.2	89.41	26.87	142.3	87620243	2.55e-24
YOLOv5L	94.08	86.75	88.83	36.62	41.3	5304534	2.84e-24
YOLOv5L-s	93.6	86.58	88.6	**101.30**	**12.3**	**1542966**	1.77e-20
YOLOv5L-s-CBAM	94.65	89.28	90.15	97.23	13.2	1564439	9.21e-17
YOLOv5L-s-SimAM	94.98	89.66	90.21	**101.30**	**12.3**	**1542966**	1.61e-13
YOLOv5L-s-SimAM-ASFF	**95.64**	**91.28**	**91.1**	95.70	17.9	2234484	1.53e-21

Bold represents the optimal values. mAP, Recall, F1, and FPS are bolded for maximum values. Size, Parameters, and p-value are bolded for minimum values.

### 3.1 Compare the performance metrics of initial models

**Figure 9:**
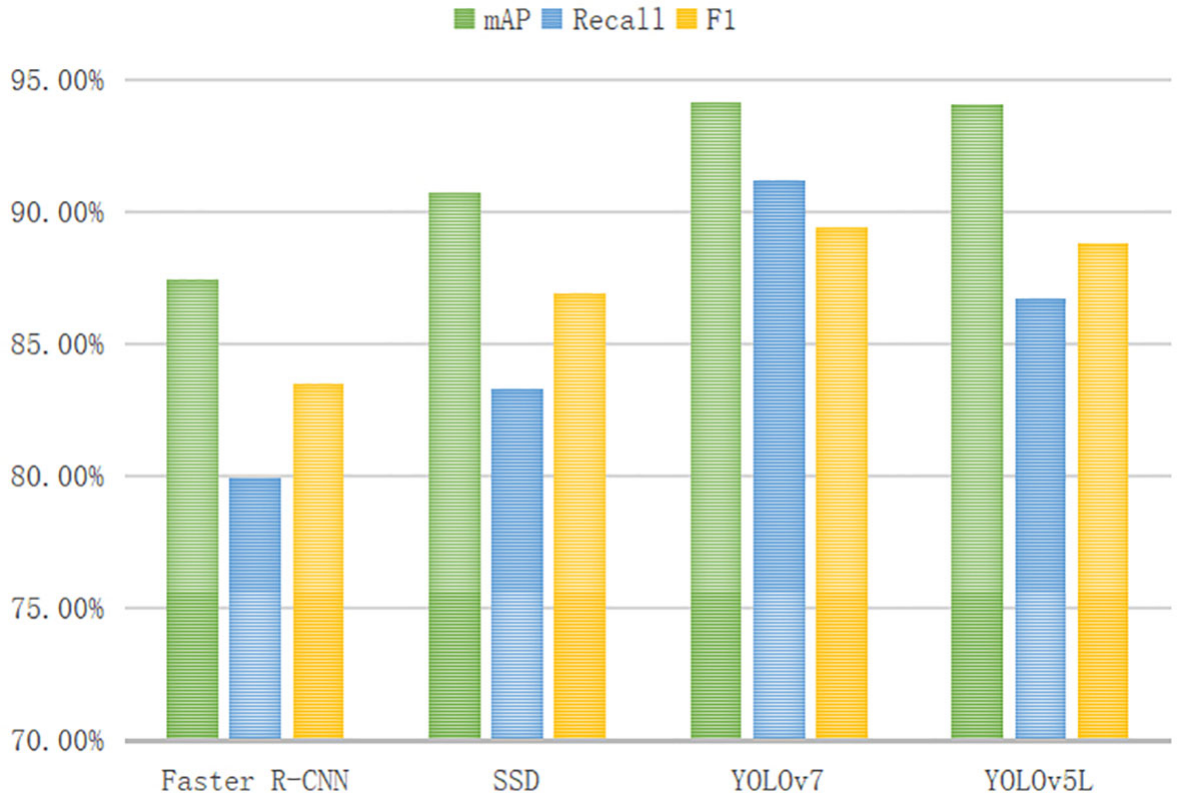
Comparison of accuracy evaluation indexes of initial models.

To intuitively compare the accuracy evaluation indexes for the four initial models of Faster R-CNN, SSD, YOLOv7, and YOLOv5L, a figure is drawn to display the heights of the vertical cylindrical bodies (as shown in **Figure 9**). The YOLO series, as more advanced models, outperform SSD and Faster R-CNN in terms of mAP, Recall, and F1 metrics, demonstrating superior performance. While the FPS values of SSD and YOLOv7 are comparable, although YOLOv7 achieves a higher mAP, it also has a larger parameter count. In terms of lightweight evaluation metrics, YOLOv5L shows more comprehensive performance metrics compared to SSD and YOLOv7.

### 3.2 Lighten backbone network of YOLOv5L

**Figure 10:**
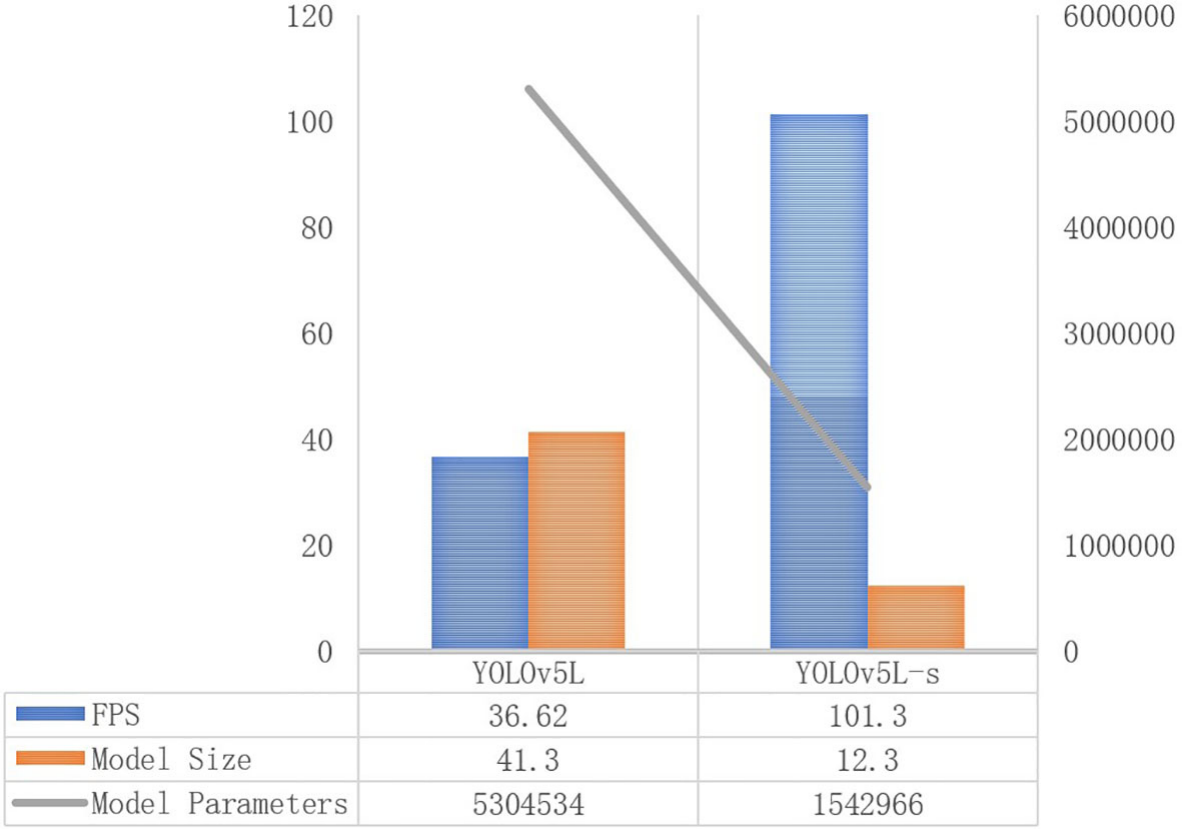
Evaluation index of different initial model accuracy.

The results indicate that although YOLOv5L-s slightly underperforms compared to YOLOv5L in terms of accuracy performance, the most significant improvement lies in the model’s lightweight characteristics (as shown in **Figure 10**). Specifically, compared to YOLOv5L, the FPS of YOLOv5L-s is improved by 2.76 times approximately, jumping from 36.62 to 101.30. Additionally, the model size of YOLOv5L-s is dropped to 12.3 MB, which is equivalent to 29% of the original YOLOv5L. Furthermore, the number of parameters of YOLOv5L-s is reduced to 1542966, which is equivalent to 29.1% of the YOLOv5L model.

### 3.3 Enhanced feature extraction of YOLOv5L-s

**Figure 11:**
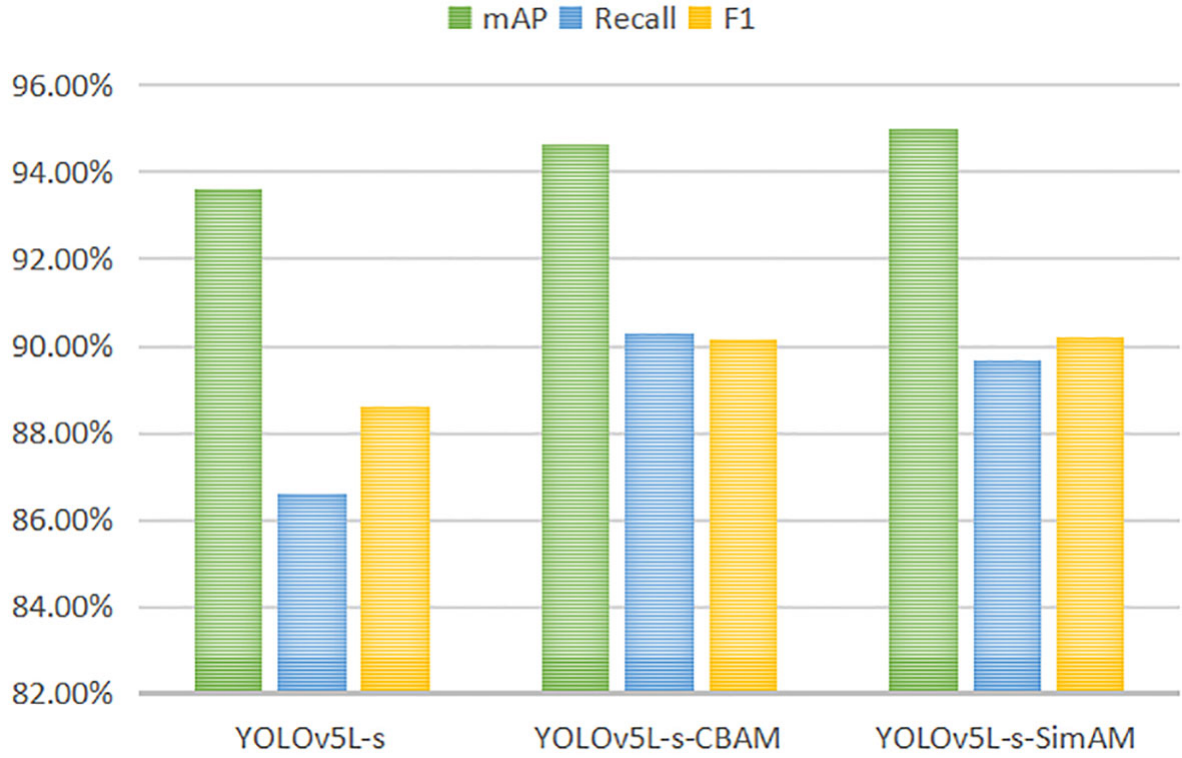
Comparison of accuracy evaluation indexes of YOLOv5L-s, YOLOv5L-s-CBAM and YOLOv5L-s-SimAM.

As shown in **Figure 11**, the mAP has an increasement of 1.38% by introducing the SimAM to the YOLOv5L-s, and a boost of 1.05% by introducing the CBAM to the YOLOv5L-s, respectively. The other two accuracy evaluation indexes, Recall and F1 score, also show improvement. In terms of model lightweight metrics, YOLOv5L-s-SimAM outperforms YOLOv5L-s-CBAM in FPS, model size, and number of parameters. Notably, YOLOv5L-s-SimAM demonstrates some improvement in accuracy evaluation metrics, including mAP, Recall, and F1, when compared to YOLOv5L-s, without any compromise in its lightweight characteristics (as shown in **Table 2**). The results indicate that the SimAM module contributes to enhancing the overall performance of the model to a certain degree.

### 3.4 Enhanced feature fusion of YOLOv5L-s-SimAM

The addition of the ASFF module after the PANet in YOLOv5L-s-SimAM further enhances feature fusion across different layers. As shown in **Table 2**, YOLOv5L-s-SimAM-ASFF achieves a remarkable mAP of 95.64% (the highest among all networks), Recall of 91.28%, and F1 score of 91.1% (also the best). Obviously, the integration of ASFF has significantly improved the detection accuracy. Although, YOLOv5L-s-SimAM-ASFF exhibits slightly inferior FPS, model size, and number of model parameters compared to YOLOv5L-s-SimAM in terms of model lightweighting, it is still significantly better than ones of the original YOLOv5L model. Comprehensively, YOLOv5L-s-SimAM-ASFF emerges as the optimal model.

**Figure 12:**
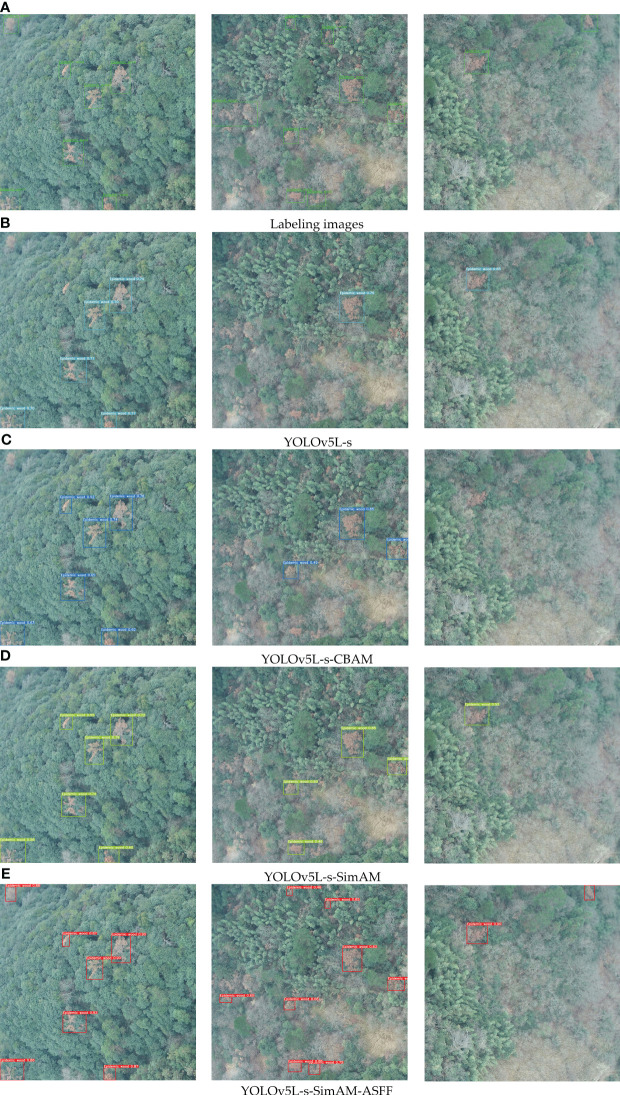
Labeling images and comparison of detection performance of the four improved models. **(A)** stands for Label image, **(B)** stands for detection result by YOLOv5L-s, **(C)** stands for detection result by YOLOv5L-s-CBAM, **(D)** stands for detection result by YOLOv5L-s-SimAM, **(E)** stands for detection result by YOLOv5L-s-SimAM-ASFF. Where, the left images show scenes with larger targets of pine wilt diseased trees and less background interference, the middle images indicate scenes with smaller targets of pine wilt diseased trees and more background interference, and the right images show scenes in which the targets of pine wilt diseased trees greatly covered by other tree species and with more complex background interference. The boxes denote the detected infected trees.

In this study, we utilized UAV images (captured by Unmanned Aerial Vehicle) with multi-resolutions and background complexities to predict pine wilt diseased trees. This approach aims to verify the detection ability of the four improved models, and partial prediction results are shown in **Figure 12**.

The detection results gradually improve from sub-figure (b) to (e). In which, the YOLOv5L-s-SimAM-ASFF demonstrates the best overall performance, especially in complex backgrounds, it effectively improves the problem of missing detection of small targets.

## 4 Discussion

Among the various detection models involved in this study, the Faster R-CNN is a two-stage network, whereas SSD and YOLO are one-stage networks. Generally, one-stage networks are structurally simpler and faster compared to the two-stage networks, making them ideal for real-time applications (Srivastava et al., 2021). They complete object detection through a single forward pass, enabling end-to-end training, and have certain advantages in detecting small objects. Usually, the detection of pine wilt disease-infected trees usually requires processing by investigators as soon as they obtain images from UAVs in the field, which demands high real-time performance. Therefore, using a one-stage network is more suitable for detecting PWD-infected trees.

Furthermore, an intriguing finding emerged: the performance of Yolov7 is slightly better than that of Yolov5L. This outcome could be attributed to the inherent complexity of YOLOv7 model, necessitating a more extensive dataset for adequate training and optimization. Conversely, YOLOv5L’s simpler structure facilitated better performance within the confines of a limited dataset, owing to its reduced set of parameters and features to learn. Additionally, based on the original YOLOv5-Lite, a serios of improved detection models with higher-accuracy, faster, or better lightweighting were developed. The improvements were implemented by three approaches: lightening the backbone network, enhancing the feature extraction, and optimizing the feature fusion. Subsequently, the improved models were tested under multiple scenarios with multi-resolutions and varying complex degrees of background. Ultimately, by introducing ShuffleNetV2, SimAM attention mechanism and ASFF module to the original YOLOv5-Lite algorithm, the YOLOv5L-s-SimAM-ASFF demonstrated the best overall performance, especially in complex backgrounds.

**Figure 13:**
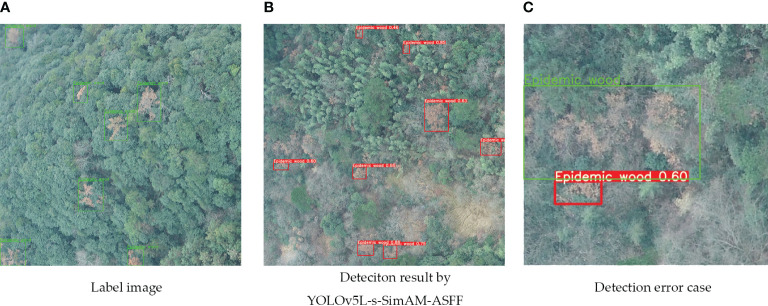
The cases of detecting errors. **(A)** stands for Label image, **(B)** stands for detection result by YOLOv5L-s-SimAM-ASFF, **(C)** stands for detection error case. Green boxes and fonts indicate the correct annotation. Red boxes and fonts indicate the result recognized by YOLOv5L-s-SimAM-ASFF.

However, despite YOLOv5L-s-SimAM-ASFF’s outstanding performance during testing, there were still shortcomings. **Figure 13** illustrates instances where trees were either not identified or incorrectly identified. Upon closer examination of **Figure 13**, we observed that the correctly annotated portions encompassed large in areas with backgrounds, including trees of various colors such as green, reddish-brown, light reddish-brown, and grayish-white. The primary reasons for the missed identifications were the subtlety of PWD features and significant interference from the surrounding environment. While the integration of ASFF enhanced the model’s capacity to detect smaller targets, it occasionally led to incorrect identifications. We suspect that this could be due to an imbalance in the number of training samples, resulting in a model that may not have been fully trained to recognize certain features or classes accurately. Moving forward, we plan to address these limitations by exploring strategies to balance the training dataset and further refine the model’s detection capabilities.

To embed the model into mobile devices in the future, it is crucial to strike a balance between lightness, accuracy, and speed. Our findings showed that by swapping YOLOv5L’s CSPDarknet53 backbone with ShuffleNetV2, we achieve significantly improvement in FPS, model size reduction, and parameter optimization.

Notably, the integration of ShuffleNetV2’s channel shuffle operation boosts the model’s feature representation, leading to an increase in mAP. Specifically, YOLOv5L-s, when compared to its predecessor YOLOv5L, experiences a significant jump in FPS from 36.62 to 101.30, which helps to satisfy the real-time detection requirements. Furthermore, YOLOv5L-s exhibits a substantial reduction in model parameters, dropping from 5304534 to just 1542966. The model size is also reduced to 12.3 MB from 41.3MB. When compared to other studies, such as the research by Huang et al (Huang et al., 2021), which based on the improved YOLOv4 with a model size of 44.2 MB for pine wilt diseased trees identification, YOLOv5L-s emerges as a more lightweight. Compared to Zhang et al. (Zhang et al., 2022a) who utilized the improved DenseNet to detect pine wilt diseased trees, YOLOv5L-s shows slightly lower accuracy, but it’s model size and number of model parameters are much smaller. The reduction of model size and number of model parameters has great significance to make the model more adaptable to low configuration running environments and can be more easily deployed on mobile devices in the future.

Moreover, further improvements of the detection accuracy of the model are keep going by introducing the attention mechanism module. The experimental results indicate that the YOLOv5L-s-CBAM and YOLOv5L-s-SimAM perform better than YOLOv5L-s by introducing the attention mechanism modules of CBAM or SimAM. These attention mechanisms enable the network to capture finer target-related details, thereby improving the model’s ability to perceive information. Qin et al (Qin et al., 2023). achieved high accuracy in detecting pine wilt diseased trees based on improved YOLOv5 combined with attention mechanisms such as CBAM. However, SimAM proves to be a more viable option in resource-constrained settings due to its smaller model size and fewer parameters.

Leveraging the strengths of YOLOv5L-s-SimAM, this study further improves the detection accuracy of the model by incorporating ASFF. This addition effectively reduce missed detections, particularly for small targets. In YOLOv5L-s-SimAM, after inputting images into the three feature layers of PANet, large targets are detected in the higher layers and small targets are detected in the lower layers. During the detection, the layer-to-layer interaction exists only for up-sampling and down-sampling operations so that many of the higher and lower layer features have not been utilized. In fact, large targets in images require larger perceptual fields and higher-level semantic features, while small targets require fine-grained features in low-level features to be discriminated. Fortunately, the ASFF can fuse features from different layers together by adaptively learning the weight coefficients of the mapping fusion of feature layers at each scale and filter the features of other layers, retaining only the useful information for that layer. This greatly enriches the model’s ability to perceive high-level semantic information and underlying detailed information of diseased pine trees. Although the model size and number of model parameters are slightly larger after the introduction of the ASFF, the detection accuracy of the model is improved significantly. As a result, the YOLOv5L-s-SimAM-ASFF emerges as the optimal model, especially adept at detecting small targets within complex backgrounds.

Owing to having both top-down and bottom-up structures, PANet network does not make better use of the semantic information of high-level features and the detailed information of low-level features. The current implementation of ASFF at the end of PANet may not fully harness the potential of high-level and low-level features. Further research could explore the strategic placement of ASFF within the PANet structure to maximize information utilization. Additionally, experiments with attention mechanisms integrated into the ShuffleNetV2 backbone could further bolster the model’s feature extraction and overall accuracy.

## 5 Conclusions

Based on our study, we propose the YOLOv5L-s-SimAM-ASFF, which is a lightweight and accurate detection model for PWD-infected trees. By combining ShuffleNetV2 as the backbone network, SimAM attention mechanism, and ASFF for multi-scale feature fusion, our model effectively improves detection accuracy while reducing computational overhead. Our experimental results show that YOLOv5L-s-SimAM-ASFF achieves optimal performance with a mAP of 95.64%, Recall of 91.28%, F1 score of 91.1%, and FPS of 95.70. These findings suggest that our model is highly suitable for real-time, high-accuracy detection of PWD-infected trees, providing valuable guidance for the identification and management of infected trees.

## Data availability statement

The raw data supporting the conclusions of this article will be made available by the authors, without undue reservation.

## Author contributions

ZD: Writing – original draft, Writing – review & editing. SW: Formal analysis, Writing – original draft. QW: Resources, Writing – review & editing. XZ: Funding acquisition, Methodology, Writing – review & editing. SL: Formal analysis, Writing – review & editing. DW: Conceptualization, Funding acquisition, Writing – review & editing.
